# Contribution of genetic variant identified in *HHEX* gene in the overweight Saudi patients confirmed with type 2 diabetes mellitus

**DOI:** 10.1016/j.sjbs.2021.10.028

**Published:** 2021-10-22

**Authors:** Mohammed Alfaifi

**Affiliations:** Department of Clinical Laboratory Sciences, College of Applied Medical Sciences, King Khalid University, Abha, Saudi Arabia

**Keywords:** HHEX, Rs7932837, T2DM, Overweight and obesity

## Abstract

**Background:**

The rs7932837 polymorphism in the Hematopoietically expressed homeobox (HHEX) gene was discovered through genome-wide association studies and is a promising candidate for type 2 diabetes mellitus (T2DM), which is one of the risk factors for obesity and other complications. T2DM has been identified as a heterogeneous and multifactorial disease characterized by insulin resistance and secretion.

**Aim:**

The aim of this study was to investigate the rs7932837 polymorphism in the HHEX gene in overweight patients diagnosed with T2DM in the Saudi Population.

**Methods:**

In this case-control study, one hundred T2DM cases and 100 controls were selected based on inclusion and exclusion criteria. Genotyping was performed with polymerase chair reaction-restriction fragment length polymorphism analysis and statistical analysis was performed between T2DM cases and controls for clinical characteristics, genotype and allele frequencies and multiple logistic regression analysis.

**Results:**

In this study, T2DM cases were compared with healthy control subjects. Clinical characteristic analysis revealed the statistical analysis between age, weight, BMI, FBG, HDL-c, TC, TG and family history (p < 0.05). HWE analysis was in the accordance (p < 0.05). The rs7932837 polymorphism in the recessive model showed the positive association (AA + AG vs AA: 2.22 [1.25–3.96] & p = 0.006) and none of the genotypes or alleles were in the statistical association. Multiple logistic regression analysis revealed positive association with age, BMI and FBG (p < 0.05).

**Conclusion:**

This study concludes as rs7932837 polymorphism in the *HHEX* gene showed positive association with recessive model and future studies recommend to carry out with large number of sample size with additional polymorphisms in *HHEX* gene.

## 1 Introduction

Hematopoietically expressed homeobox (HHEX) encodes a transcript factor that participates in the signalling of Wnt and is essential to develop ventral pancreas and liver early (Bort et al., 2004, Hunter et al., 2007). Hhex inhibits the growth of the heart, thyroid, and liver in mice. The failure to define the ventral pancreas is due to foregut endoderm cells that do not migrate as well in the uterus. Hhex activity has been found in adult mouse lungs, thyroids, and livers. Despite this, no studies have been conducted to investigate Hhex protein expression and function in the mature mouse pancreas (Zhang et al. 2014). Genome-wide association studies (GWAS) across ethnicities have frequently identified the *HHEX* gene as a plausible candidate for type 2 diabetes risk (Galavi et al. 2019). There has been a convergence of interests in searching for the genetic markers for HHEX because of the increasing prevalence of hyperglycemia. Several polymorphisms in *HHEX* gene have been discovered, with the following three variants investigated extensively: rs1111875 T > C, rs5015480 T > C, and rs7923837 A > G. Although there appears to be a correlation, the results are disputed. The difference is due to factors such as small sample numbers, incorrect selection of patients and controls, demographic stratification, and genetic backgrounds specific to distinct ethnicities (Li et al. 2012). The protein encoded by *HHEX* gene plays an important role in the initial assessment of the ventral pancreas and liver (Bhowmick et al. 2020). Despite the fact that the *HHEX* gene is located on chromosome 10q (van Vliet-Ostaptchouk et al. 2008a). Limited *meta*-analysis studies have defined the relationship between *HHEX* gene and T2DM (Cai et al., 2011, Li et al., 2012).

Diabetes mellitus (DM) is defined by persistent hyperglycemia and impaired carbohydrate, lipid, and protein metabolism as a result of total or partial insulin secretion or action (Alharbi et al. 2021a). Diabetes currently affects 463 million individuals, with 374 million suffering from poor glucose tolerance and 232 million unaware of their condition. Diabetes was directly responsible for 4.2 million deaths in 2019. Furthermore, the global cost of diabetes treatment is estimated to be around 760 billion dollars (Meo et al. 2021). In Saudi Arabia, the prevalence of T2DM has risen from 17.1% to 24.3% (Alshammari and Alnasser 2021). T2DM is characterized as a chronic condition by insulin resistance and decreased insulin production from pancreatic β-cells (Khan et al. 2015b). T2DM disease course is influenced by genetic and environmental factors. A number of behavioral and life-style factors have been associated to T2DM in both longitudinal and cross-sectional studies. An increase in the likelihood of developing insulin resistance and poor glucose tolerance has been shown in clinical and epidemiological research (Khan et al. 2015a). The majority of people with T2DM are over the age of 40, but in recent years, T2DM has been on the rise among younger populations as a result of increased inactivity, obesity, and a poor diet (Verma et al. 2021). According to prior studies, potential genes in the insulin, glucose, and adipocyte signal pathways were discovered to be involved in the pathogenesis of T2DM, and only a few polymorphisms were associated to T2DM in different ethnicities (Khan et al. 2014). T2DM has been related with various genetic polymorphism loci in the past few years, thanks to advancements in molecular biology and molecular epidemiology and the enhancement and use of gene detection technology (Cui et al. 2021).

Previous studies described the relationship between the *HHEX* gene and several types of diabetes, including T2DM, as well as *meta*-analysis studies. There were no genetic studies on T2DM patients in the Saudi population. As a result, the current study aims to evaluate the genetic relationship between the rs792387 polymorphism in the *HHEX* gene and T2DM patients in Saudi Arabia.

## 2 Materials and methods

### 2.1 Ethical statementccc

Ethical approval for this study was sanctioned from the Institutional Review Board of University Hospital. All the patients (n = 200), involved in this study has signed the informed consent form. The oral consent form has not been signed by any of the participants in this study. After receiving written informed consent, Helsinki criteria were followed for the collection of human subjects' samples (Roggli et al. 2008).

### 2.2 Sample size

The sample size for both the T2DM cases and the control subjects was calculated using an online tool named as the Survey System Creative, Research Systems. Using the aforementioned calculation, the sample size was calculated to be 25 units with a confidence level of 95% and a margin of error of 6%. Each group's sample size was calculated to be 97 persons. The total number of subjects in this study was 200, with 100 being type 2 diabetes patients and 100 being healthy controls (Hameed et al. 2021).

### 2.3 Study subjects

In this study 100 T2DM cases and 100 healthy controls were recruited in this study based on American Diabetes Association Criteria. The inclusion criteria of the T2DM cases are included if the fasting plasma glucose levels should not exceed 7.0 mmol/L. The exclusion criteria of T2DM cases were confirmed as if the normal glucose levels were obtained and the patients should be>20 years of age. The inclusion criteria for the control subjects should having normal glucose levels along without any endocrine diseases or participant should not be on any medications. The participants confirmed with any metabolic diseases or using any medication or with abnormal glucose levels were confirmed as exclusion criteria of this study. All the cases and controls were recruited from Diabetic clinic unit at King Khalid University and its hospital premises.

### 2.4 Anthropometric measurements and sample collection

The anthropometric measurements such as age, gender, weight and height were recorded in both T2DM cases and controls. Body mass index (BMI) was computed using height in centimeters and weight in kilograms (Alharbi et al. 2021b). A 5 ml blood sample was aliquoted for biochemical and molecular analysis. With the serum sample, 3 ml of biochemical sample was used for glucose and lipid profiles. The remaining 2 ml of EDTA sample was utilized for DNA isolation and molecular analysis.

### 2.5 Biochemical analysis

All 200 participants provided 3 ml of serum, which will be used to measure FBG and lipid profile parameters such as triglycerides (TGs), total cholesterol (TC), high density lipoprotein cholesterol (HDL-c), and low DL-c (LDL-c). The serum was separated by centrifuging the coagulant tube, and FBG and lipid profile values were determined using the appropriate kits.

### 2.6 Nucleotide analysis

A 600 Âµl of EDTA blood was used to extract the genomic DNA in duplicates using the Promega DNA isolation kits as per the instructions provided by the manufacturer. NanoDrop spectrophotometer was used to assess the quality and quantity of 200 DNA samples. Each DNA sample was processed to provide 10 ng of genomic DNA. Polymerase chain reaction (PCR) of the *HHEX* gene for rs7923837 polymorphism was carried out using 50 µl of reactions and 25 µl of the GoTaq master mix kit (PROMEGA, USA), which included buffer, MgCl_2_, dNTPs, and Taq DNA polymerase. The remaining products were added to the reaction mixture, including 19 µl of filtered water and 2 µl of both forward and reverse primers. To complete the reaction, 4 µl of 10 ng genomic DNA was added separately. Genotyping was carried out over 40 cycles of denaturation (94 °C-5 m), initial denaturation (94 °C-45 s), annealing (64 °C-45 s), extension (72 °C-1 m), and final extension (72 °C-10 m). Primer sequence consists of F: TGCTCACTGAACCTTGGCTA and the R: TGGCTCTTGGCCTTCTTAAA. The PCR product's band size was designed to be 222 bp, which was validated on a 2.5% agarose gel stained with 10 µl of ethidium bromide. HPY166II, a NEB restriction enzyme, was employed to cut the restriction site on the A-G rs7923837 polymorphism. Restriction Fragment Length Polymorphism (RFLP) analysis was carried out for 18 h at 37 °C to cleave the restriction site, and the following band sizes were observed after digestion: AA-222 bp, AG-222/137/85, and GG-137/85 bp, respectively.

### 2.7 Statistical analysis

The 25th version of the SPSS software was used for statistical analysis. When comparing T2DM with controls, numerical and categorical variables were examined. For the clinical data, the Student *t*-test was used to compare the two groups. Between T2DM cases and controls, all genetic models of genotypes and allele frequencies were measured. To compare genotype frequencies in controls, Hardy Weinberg Equilibrium (HWE) was used. Multiple logistic regression analysis was performed to examine the relationship between continuous and categorical factors and T2DM (Khan et al. 2019).

## 3 Results

### 3.1 Anthropmetric and clinical analysis

Table 1 shows the clinical characteristics of T2DM and healthy controls. In T2DM cases, 30% of females and 70% of male participants are present, while 42% of females and 58% of male subjects are present in controls. The mean ages of T2DM patients and controls were 55.73 ± 10.58 and 41.45 ± 8.25, respectively. T2DM had a BMI of 27.33 ± 1.78 while controls had a BMI of 25.95 ± 3.54. The weights of T2DM patients and controls were determined to be 74.34 ± 10.88 and 72.34 ± 8.54 respectively. The heights of T2DM patients and controls were found to be nearly similar (162.71 ± 8.70 vs 162.62 ± 8.61). The diabetic values were found to be 13.16 ± 5.32 for FBG and 5.22 ± 0.61 for controls. T2DM patients had HDL-c and LDL-c levels of 0.87 ± 0.38 and 3.82 ± 1.06, respectively, whereas controls had 0.67 ± 0.24 and 3.80 ± 0.82. The levels of TC and TG were found to be high in T2DM cases (5.65 ± 1.26 and 2.27 ± 1.31) and low in controls (5.19 ± 0.98 and 1.57 ± 0.71). There was a statistical relationship between family history of T2DM between cases and controls (p = 0.01). Age, weight, BMI, FBG, HDL-c, TC, and TG levels were found to be substantially associated to anthropometric and biochemical data (P < 0.05). When comparing T2DM cases and controls, other variables such as gender, height, and LDL-c levels were found to be associated (P greater than 0.05).

**Table 1:** describes the clinical features between T2DM cases and controls.

**Characteristics**	**Cases (n = 100)**	**Controls (n = 100)**	**P Values**
Gender [female/male]	30/70	42/58	0.58
Age [Years]	55.73 ± 10.58	41.45 ± 8.25	0.001
BMI [Kg/m^2^]	27.33 ± 1.78	25.95 ± 3.54	0.002
Weight [kgs]	74.34 ± 10.88	72.34 ± 8.54	0.01
Height [cms]	162.71 ± 8.70	162.62 ± 8.61	0.91
FBG [mmol/L]	13.16 ± 5.32	5.22 ± 0.61	<0.0001
HDL-c [mmol/L]	0.87 ± 0.38	0.67 ± 0.24	0.001
LDL-c [mmol/L]	3.82 ± 1.06	3.80 ± 0.82	0.87
TC [mmol/L]	5.65 ± 1.26	5.19 ± 0.98	0.01
TG [mmol/L]	2.27 ± 1.31	1.57 ± 0.71	<0.0001
Family History	36 (0.36)	21 (0.21)	0.01

### 3.2 HWE and rs7923837 analysis

In this study, only the rs7923837 polymorphism was genotyped between 100 T2DM patients and 100 controls (Table 2). The call rate of rs7923837 loci was found to be greater than 95%, which contributed to the results' dependability. The HWE analysis was carried out on control subjects and revealed a significant association (p = 0.02). In T2DM cases, the AA, AG, and GG genotypes were found to be 12%, 38%, and 50%, whereas in controls, the AA, AG, and GG genotypes were found to be 5%, 26%, and 69%. The A and G allele frequencies were found to be 0.69 and 0.31 in T2DM patients, 0.82 and 0.18 in control subjects. When T2DM cases were compared to control subjects, genotypes (AG vs AA: 0.90 & p = 0.39 and GG vs AA:0.30 & p = 0.02), dominant (AA vs AG + GG: 2.59 & p-0.07), co-dominant models (AA + GG vs AG: 0.57 & p = 0.06), and allele frequencies (G vs A: 0.48 & p = 0.002) revealed a negative association. Only the recessive model revealed (AA + AG vs AA: 2.22 [1.25–3.96] & p = 0.006) a positive association.

**Table 2:** Genotype and allele frequencies between T2DM cases and controls.

	**Cases**	**Controls**	**OR (95 %CI)**	**P Value**
AA	12 (12%)	05 (05%)	Locus	
AG	38 (38%)	26 (26%)	0.60 (0.19–1.93)	0.39
GG	50 (50%)	69 (69%)	0.30 (0.1–0.91)	0.02
AA vs AG + GG	88 (88%)	95 (95%)	2.59 (0.87–7.65)	0.07
AA + GG vs AG	62 (62%)	74 (74%)	0.57 (0.31–1.04)	0.06
AA + AG vs GG	50 (50%)	31 (31%)	2.22 (1.25–3.96)	0.006
G allele	62 (0.31)	36 (0.18)	Locus	
A allele	138 (0.69)	164 (0.82)	0.48 (0.30–0.78)	0.002

### 3.3 Multiple logistic regression analysis

Table 3 shows the results of a multiple logistic regression analysis carried between the rs7923837 polymorphism and T2DM characteristics such as gender, age, BMI, height, weight, FBS, HDL-c, LDL-c, TC, TG, family history, and AG and GG genotypes. Age (p = 0.0001), BMI (p = 0.003), and FBG (p = 0.0001) were found to have a positive correlation, while gender (p = 0.81), weight (p = 0.78), height (p = 0.95), HDL-c (p = 0.61), LDL-c (p = 0.73), TG (p = 0.69), TC (p = 0.94), AG genotype (p = 0.39), and GG genotype (p = 0.02) were found to have negative association.

**Table 3:** Multiple logistic regression analysis association with rs7923837 polymorphism in T2DM risk.

	**β**	**SE**	**OR (95 %CI)**	**P value**
Gender	0.176	0.55	1.25 (0.61–1.47)	0.81
Age	345.0	18.64	3.21 (2.14–4.32)	0.0001
BMI	567.0	13.87	1.78 (1.69–2.32)	0.003
Height	0.183	0.87	1.0 (0.96–1.0)	0.95
Weight	0.174	0.96	1.0 (0.95–1.0)	0.78
FBG	358.0	18.76	3.19 (2.16–4.28)	0.0001
HDL-c	0.089	0.22	1.0 (0.97–1.0)	0.61
LDL-c	0.071	0.31	1.0 (0.96–1.0)	0.73
TG	0.063	0.57	1.0 (0.95–1.0)	0.69
TC	0.041	0.72	1.0 (0.98–1.0)	0.94
AG Genotype	0.16	0.41	0.60 (0.19–1.93)	0.39
GG Genotype	0.32	0.48	0.30 (0.10–0.91)	0.02

AA genotype is regarded as the reference genotype.

## 4 Discussion

The aim of this study was to investigate genetic association between rs7923837 polymorphism in overweight subjects confirmed with T2DM in the Saudi population. This study included 100 T2DM cases and 100 controls, and samples were chosen based on a sample size analysis. PCR-RFLP analysis was used in this study. In this case-control study, recessive model showed the significant association when performed between T2DM cases and controls. Other genetic models of genotypes and allele frequencies didn’t show any statistical association. Multiple logistic regression analysis revealed a correlation with age, BMI, and FBG (p < 0.05), while other factors revealed a negative relation. Age, BMI, weight, FBG, HDL-c, TC, TG, and family history were all associated with T2DM when compared to controls.

In T2DM cases, age and BMI have an essential impact in the development of future complications, with hypertension, obesity, and cardiovascular disease being some of the complications revealed. Insulin treatment is another typical issue associated with weight gain and diabetes, and it has a considerable pathophysiological impact on various phases of the disease. Children who were height, overweight or obese, or had abnormal waist circumferences had higher rates of insulin resistance. Obesity, for example, arises when adiposity returns at the age of three, resulting in a higher BMI in adolescence. Obesity and insulin insufficiency are the root causes of T2DM (Chobot et al. 2018). In one of the *meta*-analysis studies concludes as Obese men had a sevenfold higher risk of T2DM than those in the healthy weight range, while obese women had a twelvefold higher risk (Guh et al. 2009). In our study, both T2DM cases (27.33 ± 1.78) and control subjects (25.95 ± 3.54) were found to be overweight. Another *meta*-analysis study indicated that regular exercise for at least 12 weeks can reduce glycated hemoglobin levels in diabetes individuals by 0.67% even in the absence of a significant reduction in BMI (Umpierre et al. 2011) and this study was also supported by the recent *meta*-analysis (Moghetti et al. 2020). Moreover, patients should focus on modifiable and non-modifiable risk factors for disease control. Saudi Arabia is one of the countries in the world where chronic diseases such as T2DM, obesity, and HTN are on the rising (Abdulaziz Al Dawish et al. 2016). T2DM prevalence was determined to be 25.7%, 16.1%, 21% and 31.6% in Bahrain, Oman, Kuwait, and Saudi Arabia, respectively. Males were reported to be 34.6% while females were found to be 27.6% within the Kingdom. When compared to Saudi Arabia, other Arab countries such as Egypt, Yemen, Iraq, and Algeria have lower frequencies such as 11.4%, 3%, 10.2%, and 8.5% (Al Mansour 2020).

The role of single nucleotide polymorphisms (SNPs) in human diseases, particularly T2DM, has been confirmed by GWAS. SNPs at more than 80 loci have been identified as risk alleles for T2DM in various populations. Variations in the genome, particularly SNPs, impact the level and function of gene expression and may alter the risk of developing T2DM (Alharbi et al., 2014, Sharif et al., 2018).

Both GWAS and *meta*-analysis studies in T2DM revealed the role of the rs7923837 polymorphism in the *HHEX* gene. In a GWAS of the French population, Sladek et al found four novel risk loci for T2DM and showed that the statistical significances of three SNPs in rs7923837 and rs1111875 in the IDE-KIF11-HHEX gene locus and rs13266634 in the SLC30A8 gene locus were very robust. A number of T2DM-related chromosomal regions have been identified through linkage studies (*CAPN10, ENPP1, HNF4A*, and *ADIPOQ*) and candidate-gene studies (*PPARG* and *KCNJ11*) coding variants being two of the few that have been effectively duplicated in a cell culture environment (Sladek et al. 2007). The *HHEX* gene is essential for pancreatic and hepatic organogenesis and is proximal to rs7923837 and rs1111875. One issue with SNP analysis is that these results do not coincide with the coding and regulatory regions of the three genes (Furukawa et al. 2008a). In both European and Japanese populations, the SNPs rs1111875 and rs7923837 have been associated to T2DM (Staiger et al., 2008, Horikawa et al., 2008). HHEX is found on chromosome 10q23.33, next to the insulin-degrading enzyme gene. Because these transcription factors may be involved in regulating insulin secretion as well as glucose and lipid metabolism, the *HHEX* gene plays important roles in carbohydrate intolerance and diabetes (Tarnowski et al. 2017).

The rs7923837 polymorphism study was conducted in many types of diabetes, including T2DM, gestational, and type 1 diabetes (Furukawa et al., 2008, Tarnowski et al., 2017, Winkler et al., 2009). Global studies, including *meta*-analyses (Cai et al., 2011, Li et al., 2012), revealed a positive and negative association with the rs7923837 polymorphism (Chidambaram et al., 2010, Fu et al., 2012, Furukawa et al., 2008, Horikawa et al., 2008, Horikoshi et al., 2007, Kommoju et al., 2016, Omori et al., 2008, Pivovarova et al., 2009, Qian et al., 2012, Sun et al., 2016, van Vliet-Ostaptchouk et al., 2008). *HHEX* gene polymorphism must be recognized as a risk factor for T2DM in obese persons. The exact structure of the HHEX protein, as well as its function and relationship to obesity, are still unknown.

This study has certain limitations as (i) only single polymorphism was screened and (ii) small sample size was enrolled and (iii) no protein studies were carried out. The strength of this study was performed with 100 of equal number of T2DM cases and controls. This study was well-designed by using specific restriction enzyme. None of the patient or control was found to be obese or morbid obese. This study was designed as similar gender-based study in both T2DM cases and controls (p = 0.58). Based on GWAS and *meta*-analysis studies, the rs7923837 SNP was opted.

## 5 Conclusion

This study demonstrates as rs7923837 polymorphism was associated with T2DM in the Saudi population with recessive mode of inheritance. None of the other genotypes or allele showed the positive association. Future studies should be designed with other polymorphisms in the *HHEX* gene, large sample size and need to involve the T2DM patients with obesity and other complications. Meta-analysis studies should be performed with all the polymorphisms in *HHEX* gene with T2DM as well as in other forms of diabetes.

## Declaration of Competing Interest

The authors declare that they have no known competing financial interests or personal relationships that could have appeared to influence the work reported in this paper.
